# Development and Validation of the Resilience in Eating Disorders Scale (RED-5)

**DOI:** 10.62641/aep.v54i1.2008

**Published:** 2026-02-15

**Authors:** Carlota Las-Hayas, Odin Hjemdal, Pedro-José Muñoz, Jesús-Ángel Padierna Acero, Luis Beato-Fernandez, Andrés Gómez-del-Barrio, Diana M. Pérez-Valencia, Amaia Pikatza-Huerga, Aitor Almeida

**Affiliations:** ^1^DeustoMED, Department of Medicine, Faculty of Health Sciences, University of Deusto, 48007 Bilbao, Spain; ^2^Red de Investigación en Cronicidad, Atención Primaria y Prevención y Promoción de la Salud (RICAPPS), 08007 Barcelona, Spain; ^3^Department of Psychology, Norwegian University of Sciences and Technology, 7034 Trondheim, Norway; ^4^Psychiatry Service, Ortuella Mental Health Center, 48530 Bizkaia, Spain; ^5^Psychiatry Service, Galdakao-Usansolo Hospital, 48960 Bizkaia, Spain; ^6^Red de Investigación en Servicios de Salud en Enfermedades Crónicas (REDISSEC), 48960 Bizkaia, Spain; ^7^Eating Disorders Unit, General Hospital of Ciudad Real, 13005 Ciudad Real, Spain; ^8^Valdecilla Biomedical Research Institute (IDIVAL), 39011 Santander, Spain; ^9^Eating Disorders Unit, Department of Psychiatry, Hospital Universitario Marqués Valdecilla, 39002 Cantabria, Spain; ^10^Centro de Investigación Biomédica en Red de Salud Mental (CIBERSAM), 28029 Madrid, Spain; ^11^DeustoTech, Faculty of Engineering, University of Deusto, 48007 Bizkaia, Spain

**Keywords:** resilience, psychometry, eating disorders, recovery, mental health

## Abstract

**Background:**

A resilience scale tailored for individuals with eating disorders (EDs) could significantly enhance our understanding and treatment of EDs. Therefore, we developed and psychometrically evaluated a new Resilience in Eating Disorders scale (RED) following COnsensus-based Standards for the selection of health Measurement INstruments (COSMIN) guidelines.

**Method:**

Informed by prior qualitative interviews, the new RED scale underwent an initial pilot test among patients with EDs (*n* = 52) and field tests among patients with EDs (*n* = 169), ED-recovered individuals (*n* = 61), and a normative sample of the general population (*n* = 349), all aged between 27.9 and 29.8 years and residing in Spain. In this study, the participants completed the RED scale, Resilience Scale-25 (RS-25), Eating Attitudes Test-26 (EAT-26), World Health Organisation Quality of Life Scale – Brief Version (WHOQOL-BREF), and Hospital Anxiety and Depression Scale (HADS). Data were collected at baseline and after 1 year. Alongside machine learning techniques, exploratory and confirmatory analyses were employed to evaluate the reliability, construct validity, convergent validity, known-groups validity, predictive validity and responsiveness of the RED scale.

**Results:**

The original 52-item version of the RED scale was refined to 44 items following the pilot phase, and ultimately reduced to a 5-item version (RED-5) after field testing and psychometric evaluation. The RED-5 demonstrated strong psychometric properties, with excellent model fit indices (Root Mean Square Error of Approximation (RMSEA) = 0.03, and Comparative Fit Index (CFI) = 0.99) and acceptable internal consistency (Cronbach's alpha = 0.71). Additionally, the RED-5 scale effectively predicted quality of life, anxiety, depression, and ED symptomatology over a 1-year period.

**Conclusions:**

The RED-5 is a concise, psychometrically robust scale specifically developed to assess resilience in patients with EDs. It significantly predicts ED symptoms and quality-of-life outcomes, making it a valuable tool for both clinical practice and research. The RED-5 allows for quick administration and easy scoring. It provides mental health professionals with a tool to guide resilience-informed assessment and more personalized treatment planning.

## Introduction

Predominantly affecting women, eating disorders (EDs) are serious mental and behavioural health conditions characterised by high mortality rates and significant psychosocial challenges [1, 2]. The fifth edition of the *Diagnostic and Statistical Manual of Mental Disorders* [3] identifies eight subtypes of EDs, with anorexia nervosa (AN), bulimia nervosa (BN), binge eating disorder (BED) and other specified feeding or eating disorder (OSFED) being the most commonly recognised.

The incidence rates of EDs (i.e., new cases within a specific time frame in a population) vary according to the methodologies, populations and diagnostic criteria used in different studies. For example, Stice *et al*. [4] observed 496 women aged 12 to 20 years across 8 years in a large US city and identified cumulative incidence rates of 0.8% for AN, 2.2% for BN, 2.7% for BED and 10.8% for OSFED. van Eeden *et al*. [5] reported lifetime prevalence rates (i.e., the percentage of the population ever diagnosed with an ED) of 4% and 3% for AN and BN, respectively, among women. However, these figures likely underestimate the true prevalence, as many cases of AN and BN go undiagnosed and unreported, partly due to these patients’ reluctance to seek help.

Despite treatment advances, a long-term follow-up study reports that only 31.4% and 68.2% of individuals with AN or BN, respectively, recovered after 9 years [6]. As a dynamic process wherein individuals’ personal skills and external social and community resources interact to enhance their mental well-being in the face of risk factors [7, 8], resilience has been identified as both a protective factor against the onset of EDs [9] and a predictor of recovery [10]. However, scholars have only employed generic resilience scales to measure resilience, such as the Connor–Davidson Resilience Scale [11] or the Resilience Scale 25 [12], due to the absence of a scale specifically designed for EDs. When targeting specific groups, disease-specific questionnaires are more advantageous than generic ones, offering focused assessments uniquely suited to the studied condition that measure individuals’ heightened responsiveness to change [13].

This study aimed to develop a new resilience scale tailored for individuals with EDs, the Resilience in Eating Disorders (RED) scale, in addition to comprehensively evaluating its validity and reliability. Based on our prior research [14, 15], we hypothesised a positive correlation between resilience levels in individuals with EDs and improved health outcomes both cross-sectionally and longitudinally. We used two methods to assess the predictive validity of the RED scale: a longitudinal analysis to determine whether resilience predicts enhanced mental health outcomes among individuals with EDs after 1 year, in addition to machine learning techniques to evaluate its predictive power in forecasting which patients will score below the threshold on an ED screening test after 1 year, based on their baseline scores.

## Materials and Methods

The Ethics Committee of the University of Deusto (Ref: Psi-01/11-12 and Psi-08/11-12; renewed approval: ETK-23/22-23) approved the present study, which also garnered support from both the research and ethics committees at the participating healthcare centres. This study complies with the 2024 Declaration of Helsinki. The development of the questionnaire rigorously followed COSMIN (COnsensus-based Standards for the selection of health Measurement INstruments) guidelines to ensure its reliability and validity in assessing the intended health outcomes [16], including better quality of life, reduced ED symptoms, and lower anxiety and depression levels. All participants provided informed consent and confirmed their voluntary participation in the study before data collection.

### Item Bank Development and Pilot Testing

The initial contents of the new RED scale were drawn from an earlier qualitative study conducted by Las Hayas *et al*. [14], which detailed experiences of resilience among individuals with EDs as narrated by individuals who had recovered from their EDs (i.e., ‘ED-recovered individuals’), their caregivers and ED specialists (e.g., psychologists and psychiatrists). This study identified several core themes associated with resilience in EDs, such as positive personality traits, personal values, motivation to change, goal setting, emotional and social support, disengagement from harmful relationships, emotional expression, mindfulness, and regulation of negative emotions.

To operationalise these themes, the first author generated an initial pool of 5–10 items per theme, either adapted from existing validated measures or newly created using the language and narratives of participants. Each item aimed to reflect a specific observable behavior, coping strategy, or psychological attitude related to resilience.

Four clinicians specializing in eating disorders—co-authors of this study—independently reviewed the item pool, selecting up to five items per theme and refining the wording to enhance clarity, relevance, and alignment with the intended constructs. This process yielded a 52-item version of the RED scale, with items rated on a 5-point Likert scale (1 = totally false, 5 = totally true), where higher scores indicate greater resilience.

To illustrate this process, one final RED-5 item—“I have had experiences where I’ve connected in a special way with my surroundings, in the here and now, and disconnected from my eating problems”—was directly inspired by participant narratives such as:

*“In specific moments of my life, hard times, very hard, a spark goes off. And that spark can be [that] I become aware of a flower and suddenly notice it, I notice… a bird… the steps of a child… it has happened to me so many times.”*

*(Excerpt from an individual interview with a participant recovered from an ED)*

The initial pilot test took place from October to November 2012 among 52 patients with EDs living in Spain (86.8% women with an average age of 28.1 years, standard deviation [SD] = 8.7), who were member of the Association Against Anorexia and Bulimia of Euskadi (ACABE). To ensure anonymity, the ACABE secretary sent private emails to the participants with details about the study’s voluntary nature and a link to the online version of the initial 52-item RED scale. Participants spent 5 to 30 minutes completing the questionnaire (average = 12.1 min, SD = 5.7), with 95.9% approving its length, 77.4% finding the wording of items understandable and 86.8% viewing the items as respectful. Open-ended feedback was also collected, including the following two examples:

1. *I found the questionnaire comprehensive and accurate. I identified closely with each question. Although more questions could precisely identify the disorder, the fundamental ones are well chosen. While I am currently battling this disease, I’ve relapsed, but continue to strive for recovery. There are many steps left, but with professional help and personal effort, I aim to live a high-quality life filled with happiness. Thank you for this tool.*

2. *I was surprised at how well the answers to the questions aligned with my feelings.*

After piloting the initial 52-item version of the RED scale, a descriptive analysis of item responses was conducted. Subsequently, the team of experts (comprising the authors of this paper) re-evaluated the content validity of all items. Based on this expert review, 11 items were removed due to one or more of the following reasons: conceptual redundancy with other items, lack of clarity or interpretability, and limited relevance to the construct of psychological resilience in the context of eating disorders.

Following this refinement, three additional items were incorporated: one assessing participants’ comprehension of the resilience construct, one capturing the individual’s self-perceived current level of resilience, and one exploring coping strategies involving engagement in pleasurable activities. These modifications resulted in a refined 44-item version used in the main field study. The Cronbach’s alpha of this version of the scale was 0.84, indicating high reliability.

### Field Study

#### Sampling and Data Collection

Data were collected from three groups: a normative sample of the general public, individuals with EDs undergoing treatment, and ED-recovered individuals, at the baseline and 1 year later. Recruitment ran from April 2013 to October 2014. Members of the general public took part in this study by completing an online questionnaire requiring their informed consent and confirmation of voluntary participation. Eligibility was limited to women aged over 18 years without a history of EDs, and participants had the opportunity to win a tablet. The study was promoted at the University of Deusto via signage, flyers and social media.

Recruitment followed a convenience method, as patients with EDs and ED-recovered individuals were recruited by psychiatrists from four public mental health centres in three Spanish regions, using DSM-IV-TR criteria for current patients and discharge status for former patients [17]. All participants were Spanish-speaking women, which defines the cultural and linguistic background of the sample. Participants could complete the questionnaire online administered through the *EncuestaFacil* platform (https://www.encuestafacil.com), by phone or on paper, with current patients receiving a gift card and former patients entering the draw to win a tablet. Participants who did not complete the questionnaire were followed up and reminded of the need to complete the questionnaire.

#### Materials

At the baseline, the participants provided their socio-demographic and clinical information, including sex, age, diagnosed ED subtype, age at ED onset, ED duration, time in treatment, discharge date, comorbidities and current psychiatric medications. The general population also answered similar clinical history questions to identify any past ED diagnosis. Race and primary language were not collected, as the majority of the participants were Caucasian and Spanish-speaking.

In addition to completing the 44-item version of the RED scale, the participants completed four standardised questionnaires:

1. Eating Attitudes Test-26 (EAT-26) [18]: This test evaluates the behavioural and cognitive symptoms experienced by individuals with EDs, providing scores from 0 to 76 (with higher scores indicating more severe symptomatology), with a cut-off of 20 for the risk of EDs. The Spanish-validated version of the EAT-26 [19] was used, with an internal consistency greater than 0.90.

2. World Health Organisation Quality of Life Scale – Brief Version (WHOQOL-BREF) [20]: Assessing general quality of life, this brief 26-item questionnaire covers physical, psychological, social and environmental domains, where higher scores indicate better quality of life. The Spanish version of the WHOQOL-BREF [21] was used, with internal consistencies ranging from 0.76 to 0.91 across domains.

3. Hospital Anxiety and Depression Scale (HADS) [22]: This 14-item tool measures seven items each for the depression and anxiety subscales. The Spanish version was used [23], with internal consistency indices indicating 0.87 and 0.86 for depression and anxiety, respectively.

4. Resilience Scale-25 (RS-25) [12]: Used to measure resilience as a positive personality trait aiding adaptation, the RS-25 includes ‘personal competence’ and ‘acceptance of self and life’. The Spanish version [24] was used, with internal consistencies of 0.92 for personal competence, 0.89 for acceptance of self and life, and 0.95 for the full scale. Scores ranged from 25 to 175, with higher scores indicating greater resilience.

#### Statistical Analyses

In this study, continuous socio-demographic and clinical data are presented as medians and interquartile ranges (IQRs), while categorical variables are shown as frequencies and percentages. Pairwise Wilcoxon rank sum tests and Kruskal-Wallis test were used to examine the differences in RED scores based on these variables. All analyses, except for Confirmatory Factor Analysis (CFA), were performed using SPSS software (v. 17.0; SPSS Inc., Chicago, IL, USA). CFA was conducted using Mplus software (v. 7.31; Muthén & Muthén, Los Angeles, CA, USA) [25]. Multiple Hierarchical Regression and Random Forest models were conducted using Python 3.12.7 and the following libraries: NumPy 2.0.2, pandas 2.2.3, scikit-learn 1.6.1, and imbalanced-learn 0.13.0. In all cases significance level was set at *p* < 0.05.

#### Reliability Analysis

Internal consistency was assessed using Cronbach’s alpha and McDonald’s omega, with thresholds of 0.70 considered acceptable [26]. Additionally, split-half reliability estimates have been included to provide a more comprehensive view of the instrument’s reliability.

#### Construct Validity Analysis

Exploratory Factor Analysis (EFA) with Principal Axis Factor (PAF) and varimax rotation was used to examine the factor structure of the RED scale using data with no missing values at baseline (n = 287). Cattell’s scree test [27] was used to guide the number of factors to be retained. Items with factor loadings ≥0.40 and cross-loadings ≤0.39 were considered interpretable. The structure identified by PAF was confirmed through CFA in the 1-year follow-up sample. Goodness of fit was assessed using the Comparative Fit Index (CFI), Root Mean Square Error of Approximation (RMSEA), and Non-Normative Fit Index (NNFI), with CFI and NNFI values above 0.90 and RMSEA values between 0.06 and 0.08 indicating acceptable fit [28].

#### Convergent Validity Analysis

Convergent validity was explored through Spearman’s correlation coefficient, assessing correlations between RED scale scores and scores for resilience (RS-25), quality of life (WHOQOL-BREF), mood disorders (HADS) and ED symptoms (EAT-26). The correlation strengths were defined as follows: ±0.80 (strong), ±0.50 (moderate) and ±0.20 (weak) [29].

#### Known Groups Validity

The known-groups validity of the RED scale was evaluated using the Kruskal-Wallis test, by contrasting median resilience scores from the RED scale between ED-recovered individuals, current patients with EDs, and the normative sample of the general population, with subsequent pairwise comparisons from the Wilcoxon rank sum test and epsilon squared as the effect size (ES). ED-recovered individuals were hypothesised to exhibit superior ED-specific resilience scores compared to the other groups.

#### Predictive Validity Analysis

Predictive validity was analysed using Multiple Hierarchical Regression analyses in the clinical sample (i.e., current patients with EDs and ED-recovered individuals). The factor scores from the RED scale at baseline were tested as a predictor of ED symptoms, anxiety, depression and quality of life at follow-up. Other predictors included the baseline scores for these variables, age at ED onset, duration of treatment, pharmacological medication status, and the RED score at baseline.

Advanced predictive analyses using machine learning methods assessed whether the baseline RED score predicted a risk for EDs (i.e., the EAT-26 cut-off point) 1 year later. A Random Forest Model with binary labels (i.e., score ≥20 = ‘Risk of ED’ or score <20 = ‘No risk of ED’) based on these EAT-26 scores was developed. Data from two time points (T1 and T2) were used, with EAT-26 scores at T2 converted into binary labels. The models were trained on 80% of the data and tested on the remaining 20%.

Prior to training, missing values, present in an average of 9.88% of the data, were imputed using mean substitution. The model’s configuration was optimised using a GridSearch procedure to identify the best combination of parameters. The final model used 100 decision trees, with a maximum tree depth of 5, and splitting criteria that allowed trees to divide when at least two cases shared the same node, with a minimum of one case per final branch.

Model performance was evaluated using the Area Under the Receiver Operating Characteristic Curve (AUROC), specificity, sensitivity and F1 score, all of which have a range of 0 to 1, where 1 is a perfect score and higher values indicate better performance and more reliable and precise decision-making capabilities. Besides, in the context of AUROC, a value close to 0.5 suggested performance akin to random chance, while values between 0.5 and 0.7 indicated some discriminative ability, and those from 0.7 to 0.9 reflected good to excellent performance [30].

#### Responsiveness Analysis

The responsiveness of the RED scale in the clinical sample was assessed using a 15-point change in RS-25 scores, corresponding to half a standard deviation (SD) at baseline, which served as the minimally important difference (MID) [31]. Norman *et al*. [31] reported that, across 33 published studies, MID estimates consistently approximated 0.5 SD, supporting the use of this threshold as a reliable indicator of meaningful change.

Accordingly, we applied this criterion to our sample of patients with eating disorders. The baseline SD of RS-25 scores in this group was 30.1, yielding an MID of 15 points. Patients were followed for one year and classified as improved (change >+15 points), unchanged (change between –15 and +15 points), or worsened (change <–15 points), based on their RS-25 score variations.

Standardised response mean (SRM), ES, and the responsiveness statistic were calculated for the RED scale, with values ≥0.80 considered large, 0.50–0.79 moderate, 0.20–0.49 small and 0.00–0.19 very small [32]. Positive values reflect standardised improvements, while negative values reflect the deterioration in the number of SDs for the baseline scores or differences in the scores.

## Results

### Sample Characteristics

Table 1 presents the socio-demographic and clinical characteristics of the three groups at the baseline and the 1-year follow-up. Across all three samples, there were no statistically significant differences in socio-demographic and clinical variables at baseline between those who responded at 1 year and those who did not. All *p*-values from the Wilcoxon signed rank tests comparing quantitative baseline characteristics between responders and non-responders were well above the conventional 5% significance threshold, ranging from 0.3411 to 0.9788.

**Table 1.** **Socio-demographic characteristics at baseline and at the 1-year follow-up of individuals currently diagnosed with EDs, ED-recovered individuals and the general population**.

	Patients with EDs		ED-recovered individuals		General population	
	Baseline	1-year follow-up	Baseline	1-year follow-up	Baseline	1-year follow-up
	n = 169	n = 123	n = 61	n = 31	n = 349	n = 167
Age (Median, IQR)	28 (15)	28 (15)	29 (13)	31 (14)	25 (11)	26 (11)
AN (n, %)	62 (38.5%)	55 (47.4%)	33 (55.0%)	18 (60%)	na	na
BN (n, %)	47 (29.2%)	25 (21.6%)	11 (18.3%)	6 (20.0%)	na	na
EDNOS (n, %)	21 (12.4%)	14 (12.1%)	7 (11.7%)	2 (6.7%)	na	na
Mix ED (n, %)	21 (12.4%)	16 (13.8%)	7 (11.7%)	2 (6.7%)	na	na
Comorbidities (n, %)	139 (82.2%)	93 (75.6%)	39 (63.9%)	22 (71.0%)	na	na
Medication (n, %)	99 (58.9%)	57 (46.7%)	14 (22.9%)	7 (22.6%)	27 (7.8%)	12 (7.2%)
Age at onset (Median, IQR)	17 (7.2)	na	17 (4)	na	na	na
Years in treatment (Median, IQR)	4 (8)	na	4 (5.5)	na	na	na
EAT-26 (Median, IQR)	31 (29.8)	31 (32)	4 (14)	4 (11.5)	3 (7)	3 (8)
RS-25 (Median, IQR)	104 (46.2)	104 (46)	137 (40)	142 (36.5)	131.5 (40.5)	133 (31)
Anxiety (Median, IQR)	12 (5)	12 (6)	7 (4)	6 (4)	6 (4.8)	6 (4)
Depression (Median, IQR)	8 (6)	9 (6)	3 (5)	3 (4)	2 (3)	2 (4)
Mental health (Median, IQR)	17 (7)	17 (7)	24 (6)	24 (5.5)	25 (4)	25 (4)
Physical health (Median, IQR)	23 (7)	23 (6)	29 (4)	30 (5)	29 (4)	30 (5)
Social health (Median, IQR)	7 (4)	7 (4)	11 (3)	11 (2.5)	12 (4)	12 (3)
Environmental quality (Median, IQR)	28.5 (7.5)	29 (7.5)	32 (5)	32 (4)	32 (6)	33 (7)

*Notes.* Age (range: 16 to 71 years). Age at onset (range: 8 to 42 years). Years in treatment (range: 0 to 29 years). Anxiety subscale of the HADS score (range: 0–21). Depression subscale of the HADS score (range: 0–21). Mental health subscale of the WHOQOL-BREF score (range: 6–30). Physical health subscale of the WHOQOL-BREF score (range: 7–35). Social health subscale of the WHOQOL-BREF score (range: 3–15). Environmental quality subscale of the WHOQOL-BREF score (range: 8–40). *Abbreviations:* IQR, interquartile range, calculated as IQR = Q3 – Q1; na, not applicable; AN, anorexia nervosa; BN, bulimia nervosa; EDNOS, eating disorder not otherwise specified; Mix ED, patients with more than one eating disorder; EDs, eating disorders; EAT-26, Eating Attitudes Test-26; RS-25, Resilience Scale-25.

Of the 564 individuals from the general population who accessed the online survey, 349 participated at baseline, with a response rate of 61.9%. The majority were women (*n* = 339) with median age of 25 years (IQR = 11). All baseline participants were contacted after 1 year, and 48% participated in the follow-up study. Although the inclusion criteria specified female participants, 7 male respondents were inadvertently included in the baseline sample. Their data were retained in the analyses under the rationale that their presence was unlikely to exert a significant influence on the overall findings. The male participants did not present relevant differences in sociodemographic data nor in mean scores compared to females.

Among the patients diagnosed with an ED, 181 agreed to participate, with 169 completing the questionnaire (response rate = 93.4%). The majority were women (95.2%), with median age of 28 years (IQR = 15).

Finally, 70 women who had recovered from an ED agreed to participate, with 61 completing the questionnaire (response rate = 87.1%). Their median age was 29 years (IQR = 13).

### Psychometric Analyses to Obtain the Five Items for the RED-5 Scale

The 44 items were initially analysed using EFA with varimax rotation in the baseline sample (*n* = 287). Prior to conducting the EFA, we evaluated the adequacy of the data using the Kaiser-Meyer-Olkin (KMO) measure and Bartlett’s test of sphericity. The KMO value was 0.86, indicating a high level of sampling adequacy and supporting the use of factor analysis (values above 0.80 are considered meritorious). Bartlett’s test of sphericity was also statistically significant (χ^2^(351) = 5142.519, *p* < 0.001), suggesting that the correlation matrix is not an identity matrix and that the variables are sufficiently correlated to warrant factor extraction. These results confirm the appropriateness of proceeding with the EFA. Cattell’s scree plot suggested retaining three factors (see **Supplementary Fig. 1**), which yielded a satisfactory factor structure comprising 27 items (Table 2). Items with factor loadings greater than 0.40 were grouped into three distinct domains. This structure showed no cross-loadings above 0.39 and accounted for 46.4% of the total variance.

**Table 2.** **Satisfactory factor structure of the 27-item version of the RED scale after performing a principal axis factor analysis. Rotated factor matrix. Full sample at baseline (*n* = 287)**.

	Factors		
	F1	F2	F3
RESI33	0.845		
RESI34	0.842		
RESI32	0.822		
RESI35	0.739		
RESI40	0.680		
RESI23	–0.677		
**RESI27**	**0.660**		
RESI25	–0.629		
RESI24	–0.628		
**RESI39**	**0.626**		
RESI26	0.615		
**RESI31**	**0.542**		
RESI29	0.498		
RESI20	0.493		
**RESI30**	**0.483**		
RESI28	0.480		
RESI15	0.473		
RESI38	0.468		
RESI19	0.450		
**RESI16**	**0.445**		
**RESI12**		**0.814**	
RESI14		0.798	
**RESI13**		**0.787**	
**RESI11**		**0.726**	
**RESI43**			**0.735**
**RESI42**			**0.716**
**RESI41**			**0.619**

*Notes.* Extraction method: Principal axis factor analysis. Rotation method: Varimax with Kaiser normalisation. The rotation converged in five iterations. The items of the RED-11 are presented in bold. The final items of the RED-5 scale are presented in bold and underlined. *Abbreviations:* RED, Resilience in Eating Disorders scale.

Subsequently, we applied CFA to assess the fit of this three-factor model using follow-up data (n = 190). However, this 27-item three-factor structure was not confirmed in the follow-up sample, indicating a need to further refine the model for stability (fit indices: χ^2^(321) = 1778.35, *p* < 0.001; RMSEA = 0.155 (90% CI: 0.148–0.162); NNFI = 0.565; and CFI = 0.602).

Adjustments based on modification indices led to a reduction in the number of items, resulting in a revised three-factor structure with 11 items (see items in bold in Table 2). This revised model demonstrated satisfactory reliability (alpha = 0.79) and fit indices: RMSEA = 0.044 (90% confidence interval [CI]: 0.014 to 0.066), NNFI = 0.95 and CFI = 0.96. The chi-square statistic was significant (χ^2^(41) = 59.649, *p* = 0.0299). Regarding the factor contents, the first factor (F1) was identified as resilience to distress, the second factor (F2) as self-knowledge and the third factor (F3) as motivation to change.

We then evaluated the convergent validity of the 11-item, three-factor structure of the RED scale (Table 3). Our analysis revealed that these three factors generally showed positive correlations with the criterion measure (i.e., RS-25) and quality of life, which were both statistically significant (*p* < 0.05). Conversely, these factors were negatively correlated with anxiety, depression and disordered eating symptoms (*p* < 0.05). Notably, the resilience to distress factor (F1) demonstrated particularly strong and satisfactorily high associations with these convergent measures, outperforming the other two subscales: self-knowledge (F2) and motivation to change (F3). Specifically, the self-knowledge subscale (F2) exhibited negligible or non-significant correlations with the convergent measures. This lack of significant associations suggests that the self-knowledge domain may not be aligned with the intended scope of the RED scale and could be considered for exclusion.

**Table 3.** **Correlation of the three-factor version (11 items) of the RED scale with convergent measures to explore criterion and convergent validity**.

	RED F1	RED F2	RED F3	RS-25	Physical health	Mental health	Social Rel.	Environment	Anxiety	Depression	EAT-26
RED F1	1	0.212*	0.460**	0.545**	0.389**	0.520**	0.399**	0.369**	–0.464**	–0.496**	–0.384**
RED F2	0.151*	1	0.096	0.268**	0.043	0.181*	0.010	0.190*	–0.124	–0.117	–0.015
RED F3	0.457**	0.205*	1	0.323**	0.219*	0.344**	0.210*	0.143	–0.224*	–0.281**	–0.271**
RS-25	0.619**	0.195*	0.303**	1	0.575**	0.750**	0.454**	0.574**	–0.489**	–0.687**	–0.294**
Physical health	0.456**	0.008	0.235*	0.577**	1	0.678**	0.471**	0.582**	–0.571**	–0.634**	–0.358**
Mental health	0.558**	0.068	0.287**	0.729**	0.705**	1	0.553**	0.580**	–0.539**	–0.746**	–0.352**
Social Rel.	0.499**	–0.044	0.231*	0.575**	0.557**	0.649**	1	0.371**	–0.397**	–0.553**	–0.292**
Environment	0.355**	0.076	0.137	0.522**	0.560**	0.587**	0.448**	1	–0.404**	–0.517**	–0.164*
Anxiety	–0.503**	0.001	–0.211*	–0.530**	–0.613**	–0.636**	–0.540**	–0.434**	1	0.587**	0.451**
Depression	–0.538**	–0.025	–0.265**	–0.693**	–0.674**	–0.771**	–0.674**	–0.523**	0.699**	1	0.330**
EAT-26	–0.482**	0.030	–0.275**	–0.360**	–0.447**	–0.467**	–0.469**	–0.189**	0.574**	0.507**	1

*Notes.* ** Correlation is significant at the 0.01 level (two-tailed); * Correlation is significant at the 0.05 level (two-tailed). Below diagonal line correlations are calculated only for the sample of patients currently diagnosed with an ED by a clinician (*n* = 167). Above diagonal correlations are calculated only for the sample of patients who scored above the cut-off of 20 in the EAT-26 (*n* = 139). RED F1: resilience to distress (ultimately becoming the RED-5 scale); RED F2: self-knowledge; RED F3: motivation to change.

We then assessed the predictive validity of the 11-item, three-factor structure of the RED scale. We conducted a hierarchical regression analysis in three stages (see Table 4 for details). Initially, the baseline value of the variable to be predicted was introduced as the primary predictor. The second stage incorporated clinical variables, including ‘age at onset’, ‘years of treatment’ and ‘pharmacological treatment’. The final stage involved a sequential analysis of the three factors. We first introduced the resilience to distress factor (F1), followed by its removal for the subsequent inclusion of self-knowledge (F2) and then repeated this process for motivation to change (F3).

**Table 4.** **Multiple hierarchical regressions to explore the predictive validity of the (11 items) RED scale in current patients with EDs and ED-recovered individuals (*n* = 113)**.

		EAT-26 T2				Anxiety T2				Depression T2			
Model stage		Std β	*R* ^2^	Δ *R* ^2^	F change	Std β	R^2^	Δ *R* ^2^	F change	Std β	R^2^	Δ *R* ^2^	F change
	1 – Baseline variable	0.720**	0.519	0.519	119.575**	0.697*	0.485	0.485	124.489**	0.715**	0.511	0.511	137.114**
	2 – Age onset	0.090	0.554	0.036	2.894*	0.017	0.494	0.008	0.695	0.057	0.533	0.021	1.958
	Years in treatment	0.084				0.004				0.062			
	Pharmacol.	0.131				0.096				0.121			
	3a – RED F1	–0.361**	0.641	0.087	25.889**	–0.207*	0.520	0.026	6.997*	–0.213*	0.558	0.025	7.080*
	3b – RED F2	0.007	0.557	0.000	0.013	–0.077	0.506	0.006	1.496	0.032	0.523	0.001	0.264
	3c – RED F3	–0.101	0.567	0.010	2.553	–0.110	0.512	0.012	3.209	–0.064	0.527	0.004	1.116

*Notes.* ** Coefficient/Test statistic is significant at the 0.01 level; * Coefficient/Test statistic is significant at the 0.05 level. EAT-26 T2 = Total score in the Eating Attitudes Test at the 1-year follow-up; Anxiety T2 = Total score in the anxiety subscale of the Hospital Anxiety and Depression scale at the 1-year follow-up; Depression T2 = Total score in the depression subscale of the Hospital Anxiety and Depression scale at the 1-year follow-up; Age onset = Age of the patient when the eating disorder started; Pharmacol. = Dichotomous variable for patients reporting currently taking or not taking psychiatric medication; RED F1: resilience to distress (ultimately becoming the RED-5 scale); RED F2: self-knowledge; RED F3: motivation to change. The first column outlines the model stages and the variables included at each stage; stage 3 consists of a sequential analysis of factors F1 (stage 3a), F2 (stage 3b), and F3 (stage 3c), where each factor was first introduced into the model and subsequently removed.

The standardised β coefficient revealed that the resilience to distress factor (stage 3a) significantly predicts the symptoms of EDs at follow-up (β = –0.361, *p* < 0.001), accounting for an additional 9% of variance in these symptoms. This implies that lower levels of resilience at baseline are correlated with higher symptoms at the follow-up examination. This significant relationship was also observed between the resilience to distress factor (F1) and both anxiety and depression at follow-up. Furthermore, the mental health component of the WHOQOL-BREF at follow-up (see **Supplementary Table 1**) was also significant, indicating a positive correlation between higher resilience at baseline and better mental health perceptions at follow-up (β = 0.233, *p* < 0.001), explaining nearly 3% of the variance (ΔR^2^ = 0.031, *p* < 0.001).

However, the other two subscales did not demonstrate significant predictive values for any outcomes at follow-up. Consequently, these subscales were deemed irrelevant and were subsequently excluded from the RED scale.

Based on these findings, the RED scale was streamlined to include only F1, ‘resilience to distress’. Consequently, the scale was renamed RED-5 to reflect its composition of five items. This refinement was driven by F1’s consistently robust performance across the metrics for construct validity, convergent validity and predictive validity.

In summary, the item reduction process from the initial 44 items to the final 5 was conducted in several stages. Seventeen items were removed due to low factor loadings in the EFA. Sixteen additional items were excluded based on modification indices from the CFA, indicating poor model fit. Three items were removed due to insufficient convergent validity with related constructs, including quality of life, resilience, anxiety, depression, and eating disorder symptoms. Finally, three items were discarded because they demonstrated poor predictive value for any outcomes at follow-up. A **Supplementary Table 2** provides a detailed overview of all items, including their wording and the rationale for their removal or retention.

When further investigating the psychometric properties of the RED-5 scale, we undertook a renewed analysis of its construct validity by examining it separately from its previously associated factors. We also evaluated its reliability, discriminant validity and predictive validity using advanced machine learning techniques for an in-depth assessment.

### Construct Validity of the RED-5 Scale

For the construct validity of the RED-5 scale, we conducted a second CFA on its unidimensional structure using the full baseline sample (*n* = 378). The results showed a chi-square value of χ^2^(5) = 6.662, with a non-significant *p*-value of 0.247, suggesting no significant discrepancy between the proposed model and the observed data. Although reporting fit indices is not customary when the chi-square value is non-significant, they are included here to provide comprehensive insights (i.e., RMSEA = 0.030, NNFI = 0.98 and CFI = 0.99).

### Reliability of the RED-5 Scale

Regarding the reliability of the RED-5 scale, the Cronbach’s alpha coefficient was 0.71 and McDonald’s omega of 0.72, indicating acceptable internal consistency. Additionally, split-half reliability estimates provide a more comprehensive view of the instrument’s reliability. The results for the split-half reliability estimates show a maximum value of 0.78, an average estimate of 0.69, and a minimum value of 0.64, supporting acceptable internal consistency.

### Known-Groups Validity of the RED-5 Scale

**Fig. 1.**
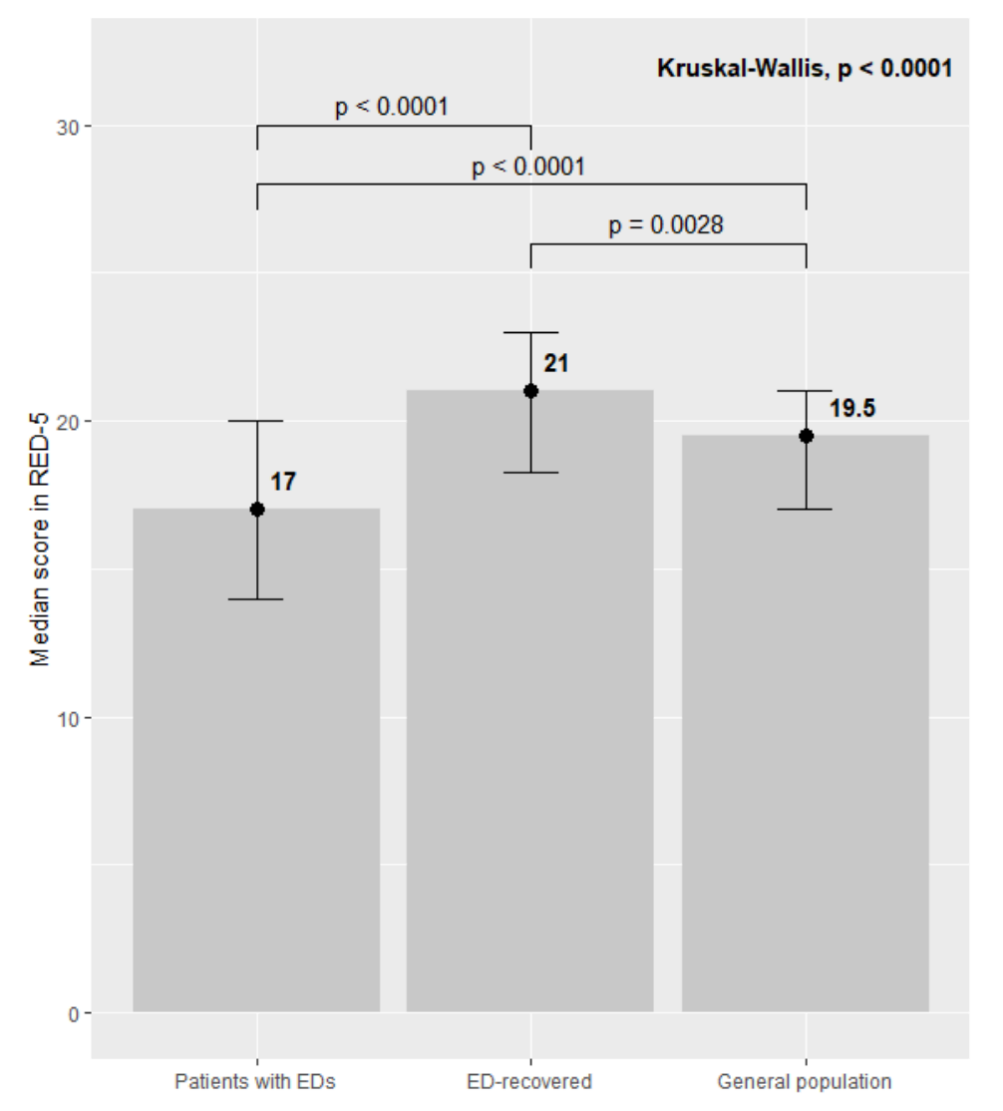
**Median scores with IQR bars for the 5-item RED scale in three different samples: patients currently diagnosed with EDs, ED-recovered individuals and a normative sample from the general population**. *p*-values from Kruskal-Wallis test and pairwise Wilcoxon tests are indicated with statistically significant differences (*p* < 0.05) between patients with EDs and each of the other two groups. Responsiveness of the RED-5 Scale.

The known-groups validity of the RED-5 scale is presented visually in Fig. 1, which displays the median scores and IQRbars for the RED-5 scale (i.e., scores ranging from 5 to 25) at baseline across three different samples: current patients with EDs (Median = 17, IQR = 6), ED-recovered individuals (Median = 21, IQR = 4.75) and the general population (Median = 19.5, IQR = 4). Kruskal-Wallis test and pairwise Wilcoxon sum rank tests revealed that the mean scores were statistically significantly different among all three groups (*p* < 0.05), with ES = 0.135 (moderate).

Over a year, the RED-5 scale demonstrated varied responsiveness indices in different patient groups, based on the RS-25 criterion for resilience. In patients who exhibited a decline in resilience, the RED-5 scale showed negative and small responsiveness indices (ES = –0.032, SRM = –0.40 and responsiveness statistic = –0.35). For patients whose resilience levels remained stable, the RED-5 scale indicated no significant changes in score magnitudes (ES = 0.14, SRM = 0.17 and responsiveness statistic = 0.19). In contrast, for patients who showed improvement in resilience, the RED-5 captured positive changes with moderate magnitude (ES = 0.57, SRM = 0.71 and responsiveness statistic = 0.70).

### Predictive Validity of the RED-5 Scale

When combined with the Random Forest model, the RED-5 scale achieved an AUROC of 0.67, which indicates the moderate capability of the model in differentiating between patients with EDs who continued to present a risk of EDs (scores of ≥20 on the EAT-26 after 1 year of follow-up) and those without such a risk (as indicated by scores <20 on the EAT-26 after 1 year). The 5-items of the RED-5 scale were entered as separate features in the model.

Using the Random Forest approach, the RED-5 scale demonstrated a specificity of 0.98 and a sensitivity of 0.35. This high specificity suggests that the model is effective in accurately identifying individuals who will have no risk of EDs 1 year later, thereby minimising the likelihood of falsely categorising individuals as symptomatic when they are not.

However, the sensitivity of 0.35 indicates that the model correctly identifies only 35% of true positive cases, which reflects a relatively low rate of detecting actual instances of EDs. With a value of 0.48, the F1 score signifies a moderate equilibrium between precision and sensitivity.

## Discussion

The current study introduced and evaluated the RED-5 scale as a new unifactorial scale for assessing resilience in patients with EDs across clinical and research settings. This development drew upon insights from ED-recovered individuals, caregivers and experts in the field. Employing a longitudinal approach and advanced machine learning techniques, this study assessed the RED-5 scale’s psychometric properties and predictive validity across diverse samples, including current patients with EDs, ED-recovered individuals and the general population. The RED-5 scale comprises a concise 5-item Likert scale (from 1 = Totally false to 5 = Totally true), where a higher score signifies a greater level of resilience. The results confirm the RED-5 scale’s validity, reliability, sensitivity to change and strong correlations with quality-of-life domains.

Although the study of resilience has proliferated since the 1970s, there is still no universally accepted definition of the construct [33]. This lack of consensus poses challenges for asserting that any specific set of items fully encapsulates the construct. However, drawing from recent conceptual updates [33, 34] and based on our previous qualitative study on the experience of resilience according to ED patients [14], we argue that the five items retained in the RED-5 scale meaningfully capture essential dimensions of psychological resilience, particularly as experienced in the context of eating disorders.

Taken together, these five items of the RED-5 cover two core components of resilience identified in recent literature: affect regulation [33] and psychological flexibility [35]. Affect regulation involves both coping responses [36] and emotional regulation, while psychological flexibility refers to the capacity for value-driven behavioral adaptation under stress. The RED-5 scale frames resilience as a dynamic, individual-level capacity that evolves across the illness trajectory. It is not assessed as a fixed trait or an outcome, but as an active process that enables reductions in psychopathology, improvements in mental well-being, and enhanced social functioning.

As outlined by Folkman and Lazarus [36], the transactional model of stress elucidates emotion-focused coping methods. According to this model, psychological stress occurs when individuals perceive a situation as exceeding their coping capabilities. To manage their stress, individuals must use coping strategies that can be problem focused, aimed at directly tackling the stressor, or emotion focused, intended to manage the associated emotional distress. An adaptive coping response effectively reduces stress and preserves well-being, whereas a maladaptive response results in adverse outcomes.

Gross [37] conceptualised emotion regulation as the management of emotional experiences and expressions, while Mallorquí-Bagué *et al*. [38] found that individuals with EDs face greater challenges in emotion regulation than healthy controls, noting that effective treatment outcomes are often parallelled by significant improvements in emotion regulation. This highlights the critical role of emotion regulation in recovering from EDs.

Three of the five items on the RED-5 scale (see Appendix Table 5) exemplify the use of adaptive emotion-focused coping methods and alternative methods for emotion regulation. In particular, the first item, ‘When I get nervous, I engage in activities that distract me (e.g., going for a walk, reading a book, talking to someone) to avoid thinking about food’, refers to using distraction as an emotional regulation strategy to deal with feelings of nervousness. Distraction is commonly viewed as a non-adaptive strategy to sidestep emotions [39]. In our study, however, distraction functions as a problem-solving strategy (i.e., a cognitive–behavioural strategy directed to eliminate stress by modifying the situation that produces it) [40]. When individuals with EDs experience nervousness, it frequently leads to ED behaviours, such as food restriction, excessive exercise or binge eating, as a means to avoid their uncomfortable emotions. Instead, a method for patients with EDs to cope with their negative emotions in a resilient way is to engage in alternative activities, such as walking or reading. Using this strategy consistently might weaken and ultimately break the relationship between negative emotions and ED behaviours. In these cases, distraction serves as a strategic problem-solving method, enabling negative emotions to be managed constructively [40]. The second item included in the RED-5 scale, ‘I have developed behaviours or activities that allow me to release my anxiety’, also reflects the adoption of a problem-solving coping strategy. This approach involves executing a planned action when the individual experiences anxiety. The third item, ‘Emotionally venting (e.g., with a friend, therapist, family member, writing) helps me to overcome my problems’, refers to emotional venting as an emotion-focused coping strategy. There are mixed results regarding the effectiveness of emotional venting as an adaptive strategy. While some studies suggest that emotional venting is associated with improved psychological outcomes [41], particularly when it occurs with a listener who helps reframe the individual’s cognitions—such as a therapist [42, 43]—others report the opposite, especially when venting escalates into rumination [44, 45]. However, a study [46] focusing on international students without robust social networks in their new country found that in situations with limited emotional support, emotional venting can be an adaptive strategy with positive psychological outcomes. This finding may also extend to patients with eating disorders, who often lack a substantial network of friends or supportive figures and tend to engage in emotional venting primarily with close contacts, including their therapists.

The fourth item on the RED-5 scale, ‘I have had experiences where I’ve connected in a special way with my surroundings, in the here and now, and disconnected from my eating problems’, describes moments of deep awareness of the environment and the present moment. This engagement with the moment helps individuals detach from their concerns related to EDs. This reported experience is aligned with the experience of mindfulness practice, where individuals pay attention to the present moment non-judgementally [47]. A study by Osborne *et al*. [48] found that individuals with mindfulness skills tended to have fewer difficulties regulating their emotions with less negative affect. Supporting these findings, using the RED-5 scale revealed that mindfulness plays an essential role as a resilience tool for individuals recovering from EDs. Finally, the fifth RED-5 item, ‘Currently, I remind myself of my own personal values to overcome difficulties’, exemplifies the concept of psychological flexibility, which Doorley *et al*. [49] described as the propensity to adapt to situations in ways that support the achievement of valued goals. Psychological flexibility entails the skill to transform negative stimuli from challenging circumstances without compromising one’s pursuit of what is genuinely valued and desired. Psychological flexibility is a critical attribute in forming resilient responses [34]. Within the frameworks of regulatory and psychological flexibility, the RED-5 scale views resilience in EDs as the enactment of context-sensitive and flexible coping strategies. This approach recognises that effective coping in the face of ED-related adversities is not about adhering to a rigid set of adaptive responses. Instead, it emphasises the importance of versatile coping mechanisms that work in tandem with psychological flexibility.

The RED-5 scale distinguishes itself from other resilience measures through its focused assessment of resilience in patients with EDs. While a variety of resilience measures are available (for a thorough review, see Windle [50]), the RED-5 scale specifically encapsulates the aspects of resilience most relevant to patients with EDs, as reported by ED-recovered individuals. This approach is a departure from using generic resilience measures or those designed for different target populations. Furthermore, our methodology adopted a variable-focused analysis of resilience, shifting away from the person-focused approach typically used in the study of resilience in ED, as detailed by Hildon *et al*. [51]. This method involves assessing the variables (such as immediate actions, iterative processes or habitual/routine behaviours) that are triggered in response to significant adversity to achieve resilient outcomes. Hildon *et al*. [51] emphasised that this type of analysis can uncover practices or assets that are particularly effective in challenging conditions, potentially buffering, transforming or mitigating the full impact of psychosocial and health-related crises.

Furthermore, we comprehensively examined the psychometric qualities of the RED-5 scale, providing practical insights for its future application in both research and clinical settings. In terms of discriminant validity, the RED-5 scale yielded higher scores in the ED-recovered sample, followed by the normative sample, with the lowest scores observed in those currently undergoing treatment for EDs. Although the difference in mean scores between the recovered and normative samples was small, it was statistically significant. The higher scores in the ED-recovered sample could be attributed to the development of the RED-5 scale based on their accounts of resilience and its specific design for participants with EDs, making its content on resilience potentially less representative for the normative (i.e., ‘healthy’) sample. The discriminant capability of the RED-5 scale bolsters our confidence in its ability to differentiate among participants with or without EDs.

The predictive validity of the RED-5 scale was also evaluated using two methods: hierarchical regression analyses and machine-learning algorithms. The results of the hierarchical regression analysis indicated that resilience, as measured by the RED-5 scale, predicted various health-related outcome measures (e.g., fewer ED symptoms, less anxiety and depression) in a 1-year follow-up, even after including the relevant clinical variables in EDs as predictors. This finding robustly supports the predictive validity of the RED-5 scale. The application of machine learning techniques yielded only moderately satisfactory results, with a moderate AUROC score of 0.67, which was likely influenced by the limited number of items in the RED-5 scale. While a questionnaire condensed to five questions may restrict the scope of information gathered, its efficiency, reduced participant fatigue and lower cost justify its use. However, it is crucial to underscore that the predictions generated by the Random Forest model should not be interpreted as definitive diagnostic outcomes. In particular, although the model achieved a high specificity of 0.98—indicating strong performance in ruling out low-risk cases—it also exhibited a notably low sensitivity (0.35), which means it failed to detect approximately two-thirds of truly at-risk individuals. This low sensitivity limits its utility for identifying high-risk patients and could lead to false reassurance if used as a stand-alone screening method. Clinicians should therefore be cautious in over-relying on negative results. The model should only be considered an auxiliary tool to support—but never replace—clinical judgement and comprehensive assessment procedures. Its outputs must always be interpreted in context, alongside other clinical indicators. Future research should continue exploring the predictive role of resilience in positive ED-related outcomes and seek to enhance the model’s performance by integrating additional clinical or psychosocial variables that may improve sensitivity without compromising specificity.

Content validity of the RED-5 was ensured through a rigorous and theory-driven development process. The scale’s initial items were derived from a comprehensive qualitative study involving individuals in clinical and functional recovery from eating disorders, as well as experienced clinicians and primary caregivers. This foundational study [14] enabled us to ground the items in lived experiences of resilience to EDs. Thematic analysis guided item generation, and themes were translated into questionnaire items with attention to clarity and singularity of construct. Existing validated resilience and mental health instruments were also consulted to align language and structure. Expert review by clinical psychologists, psychiatrists, and psychometricians—co-authors of the present paper—further ensured the scale’s content relevance and conceptual fidelity. The 52-item version of the RED was then piloted with ED patients, demonstrating strong content validity, which was a key step in the iterative refinement process that led to the final RED-5.

While the results for the RED-5 scale are satisfactory, this study acknowledges certain limitations that call for further research. One of the limitations of the questionnaire is that content evaluation was conducted by the expert panel composed of the authors, which may introduce assessment bias. Involving independent external experts could have strengthened the evaluation of the scale’s content validity. Also, the number of patients with EDs (encompassing both current and recovering patients with EDs) recruited for the follow-up study is at the lower threshold for conducting a robust CFA. Generally, recruiting a large sample of patients with EDs for longitudinal studies poses a significant challenge in ED research. Additionally, we did not evaluate the test–retest reliability of the RED-5 scale, which would provide a more comprehensive insight into its reliability. This decision was made to avoid overburdening participants, as the sample included individuals with eating disorders—a particularly vulnerable population. Another limitation of this study is that only female participants were included in the development and validation of the RED-5 scale. Although eating disorders are more prevalent among women, the exclusion of male participants restricts the generalizability of the findings and the applicability of the scale to the male ED population. Future studies should aim to include a more diverse sample, particularly men, to ensure the broader validity and relevance of the scale across genders.

Future research could employ the RED-5 scale as an effective instrument to predict future health outcomes in patients with EDs. In addition, the studied sample was relatively old and had already received therapy for many years, which could influence the life experience of the evaluated individuals and may not be generalisable to younger people or those who have not received treatment. Additionally, there was a high presence of comorbidities (e.g., personality disorder, or depression). To address the model’s current limitations, future studies should consider recalibrating the decision threshold, testing alternative machine learning algorithms, and incorporating additional risk indicators—such as clinical history, comorbid psychopathology, or behavioural markers—to increase detection capacity. Finally, it would be worthwhile to investigate whether enhancing resilience levels in patients with ED through clinical psychotherapy [52] can aid in preventing relapse episodes and improving recovery rates within this population.

## Conclusions

A new disorder-specific resilience scale for individuals with EDs, the RED-5, was introduced and validated through a comprehensive psychometric evaluation. The instrument demonstrated strong validity, reliability, and responsiveness in assessing resilience among ED populations. It captures key aspects of resilience relevant to ED recovery that are significantly associated with improved mental health outcomes, reduced ED symptomatology, and enhanced quality of life. While its predictive sensitivity was moderate, the scale serves as a valuable complementary tool to clinical judgment, enabling more individualized care in both research and clinical settings. In summary, the RED-5 is a concise, targeted, and psychometrically robust instrument that supports mental health professionals in evaluating resilience among individuals with EDs. It represents a meaningful step toward improving personalized treatment strategies and deepening our understanding of protective factors in ED recovery.

## Availability of Data and Materials

The data supporting this study’s findings are available upon request from the corresponding author and are not publicly accessible due to privacy or ethical constraints.

## Supplementary Material

Supplementary material associated with this article can be found, in the online version, at https://doi.org/10.62641/aep.v54i1.2008.

## Appendix

See Table 5.

**Table 5.** **The Spanish version of the RED-5 scale**.

Esta pregunta quiere evaluar si relacionas la comida con determinados estados de ánimo, o no, o si estás haciendo cambios en la relación entre tu estado de ánimo y tu forma de alimentarte. Por favor, responde cómo de verdadera es esta afirmación para ti:					
	Totalmente cierto	Mayormente cierto	Ni cierto, ni falso	Mayormente falso	Totalmente falso
1. Cuando me pongo nerviosa hago alguna actividad que me distraiga (p.e., salir a pasear, leer un libro, hablar con alguna persona) para evitar pensar en la comida.	5☐	4☐	3☐	2☐	1☐
Esta pregunta quiere evaluar si recientemente has experimentado momentos vitales en los que has desconectado de la enfermedad, donde no tienes pensamientos relacionados con la comida, y tienes experiencias de disfrute con la mera observación del entorno, o simplemente haciendo una actividad agradable que te desconecta de todo. Por favor, responde cómo de verdadera es esta afirmación para ti:					
	Totalmente cierto	Mayormente cierto	Ni cierto, ni falso	Mayormente falso	Totalmente falso
2. He tenido experiencias en las que he conectado de una manera especial con el entorno, en el aquí y el ahora, y he desconectado de mis problemas alimenticios.	5☐	4☐	3☐	2☐	1☐
Estas preguntas quieren conocer si en general te desahogas emocionalmente, descargas tus emociones, bien gracias a la compañía de alguien o bien escribiendo, dibujando, o expresando tus emociones de forma que las liberas. Por favor, responde cómo de verdadera es cada una de estas afirmaciones para ti:					
	Totalmente cierto	Mayormente cierto	Ni cierto, ni falso	Mayormente falso	Totalmente falso
3. Desahogarme emocionalmente (i.e., con un amigo, terapeuta, familiar, escribiendo) me ayuda a superar mis problemas	5☐	4☐	3☐	2☐	1☐
4. He desarrollado conductas o actividades que me permiten desahogar mi ansiedad.	5☐	4☐	3☐	2☐	1☐
Esta pregunta valora si para hacer cambios en tu vida has activado algunos rasgos de tu personalidad que hacía tiempo que no manifestabas, o bien has hecho cambios en tu vida defendiendo algunos valores tuyos personales, que hacía tiempo que no expresabas. Por favor, responde cómo de verdadera es esta afirmación para ti:					
	Totalmente cierto	Mayormente cierto	Ni cierto, ni falso	Mayormente falso	Totalmente falso
5. En la actualidad recuerdo mis propios valores personales para superar las dificultades.	5☐	4☐	3☐	2☐	1☐
