# Supplementary material for: Development and Validation of the Resilience in Eating Disorders Scale (RED-5)
Source: Actas Esp Psiquiatr. 2026 Feb 15;54(1):1–16. doi: 10.62641/aep.v54i1.2008 (PMC12946729; doi:10.62641/aep.v54i1.2008)
Supplement: Supplementary file 1 [file ActEsp-54-1-1-16-s1.zip › Supplementary_material.pdf]

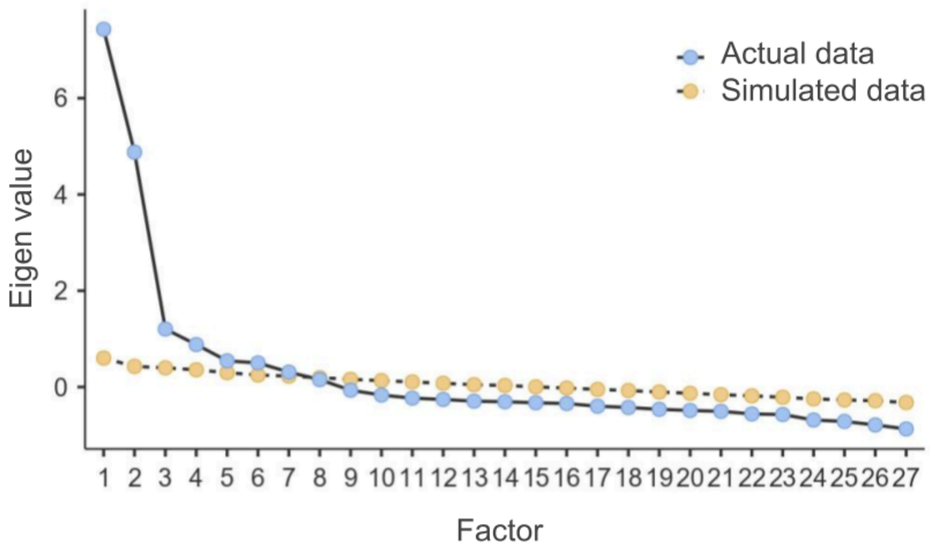

Supplementary Fig. 1. Cattell's scree test used to guide the number of factors to be retained from the EFA. The elbow point suggests a three-factor solution, as the first three factors have eigenvalues above 1.

Supplementary Table 1. (Cont.) Multiple hierarchical regressions to explore the predictive validity of the (11 items) RED scale in current patients with EDs and ED-recovered individuals (*n* = 113).

Model stage	Mental Health WHOQOL-BREF T2			
	Std β	R^2	ΔR^2	F change
1 – Baseline variable	0.816**	0.615	0.615	235.8**
2 – Age onset	0.070	0.593	0.022	1.704
Years in treatment	0.085			
Pharmacol.	1.31			
3a – RED F1	0.233**	0.624	0.031	4.969*
3b – RED F2	0.005	0.589	0	0.014
3c – RED F3	−0.093	0.591	0	0.608

Notes. ** Coefficient/Test statistic is significant at the 0.01 level; * Coefficient/Test statistic is significant at the 0.05 level. Mental Health WHOQOL-BREF T2 = Total score in the the mental health component of the WHOQOL-BREF at the 1-year follow-up; Age onset = Age of the patient when the eating disorder started; Pharmacol. = Dichotomous variable for patients reporting currently taking or not taking psychiatric medication; RED F1: resilience to distress (ultimately becoming the RED-5 scale); RED F2: self-knowledge; RED F3: motivation to change. The first column outlines the model stages and the variables included at each stage; stage 3 consists of a sequential analysis of factors F1 (stage 3a), F2 (stage 3b), and F3 (stage 3c), where each factor was first introduced into the model and subsequently removed.

Supplementary Table 2. From Red-44 to RED-5: Summary of item exclusion rationale of retained items.

Item Code	Item Text	Decision / Rationale
RESI1	If we define resilience as "an internal source of energy that motivates a person to grow or fight to overcome their problems," would you say you have experienced resilience in your struggle against the eating disorder?	Excluded due to low factor loading (< 0.40) in EFA
RESI2	If we define resilience as "an internal source of energy that motivates a person to grow or fight to overcome their problems," how would you rate your current level of resilience?	Excluded due to low factor loading (< 0.40) in EFA
RESI3	I believe I need to make drastic changes in my lifestyle because I want to improve it.	Excluded due to low factor loading (< 0.40) in EFA
RESI4	If I could live my life again, I wouldn't change much.	Excluded due to low factor loading (< 0.40) in EFA
RESI5	I feel I need a significant change in my life.	Excluded due to low factor loading (< 0.40) in EFA
RESI6	I've had a meaningful experience that made me reconsider my lifestyle.	Excluded due to low factor loading (< 0.40) in EFA
RESI7	I've experienced a turning point that changed how I see myself and my attitude toward life.	Excluded due to low factor loading (< 0.40) in EFA
RESI8	I've had a life changing experience that made me feel ready for a major change.	Excluded due to low factor loading (< 0.40) in EFA
RESI9	I am aware of the negative consequences that my eating behavior has on my own life.	Excluded due to low factor loading (< 0.40) in EFA
RESI10	I am aware of the negative consequences that my eating behavior has on others.	Excluded due to low factor loading (< 0.40) in EFA
RESI11	I could explain why I developed the eating disorder; I know its origin.	Removed due to insufficient convergent/predictive validity (originally part of Factor 2 in RED-11)
RESI12	I know at least one major reason that led me to develop an ED.	Removed due to insufficient convergent/predictive validity (originally part of Factor 2 in RED-11)
RESI13	Overall, I am aware of the reasons I've had difficulties in life.	Removed due to insufficient convergent/predictive validity (originally part of Factor 2 in RED-11)

Item Code	Item Text	Decision / Rationale
RESI14	I have a clear view of my past and how it has influenced my behavior and life choices.	Removed due to poor model fit (CFA modification indices)
RESI15	I’ve learned to do other things to distract myself when I feel bad instead of turning to food or avoiding eating.	Removed due to poor model fit (CFA modification indices)
RESI16	When I feel nervous, I do something to distract myself (e.g., walking, reading, talking) to avoid focusing on food.	Retained in RED-5
RESI17	I’ve considered starting or resuming something that excites me.	Excluded due to low factor loading (< 0.40) in EFA
RESI18	I believe it’s a good time to begin activities that align with my personal interests.	Excluded due to low factor loading (< 0.40) in EFA
RESI19	I have someone I trust to share my fears and concerns.	Removed due to poor model fit (CFA modification indices)
RESI20	I believe I have someone who supports and encourages me.	Removed due to poor model fit (CFA modification indices)
RESI21	I am the only person who can change my life.	Excluded due to low factor loading (< 0.40) in EFA
RESI22	I need to help myself, even if it creates conflict with others.	Excluded due to low factor loading (< 0.40) in EFA
RESI23	I feel sad or depressed.	Removed due to poor model fit (CFA modification indices)
RESI24	I feel irritable or angry.	Removed due to poor model fit (CFA modification indices)
RESI25	I feel tense or panicky.	Removed due to poor model fit (CFA modification indices)
RESI26	I’ve experienced moments where ED thoughts disappeared.	Removed due to poor model fit (CFA modification indices)
RESI27	I’ve connected with the present moment and disconnected from ED.	Retained in RED-5
RESI28	I’ve found myself doing things without focusing on negative thoughts.	Removed due to poor model fit (CFA modification indices)
RESI29	I have someone/something I can emotionally vent to.	Removed due to poor model fit (CFA modification indices)
RESI30	Venting emotions helps me overcome difficulties.	Retained in RED-5

Item Code	Item Text	Decision / Rationale
RESI31	I've developed ways to release my anxiety.	Retained in RED-5
RESI32	I accept myself as I am	Removed due to poor model fit (CFA modification indices)
RESI33	I accept my strengths and weaknesses.	Removed due to poor model fit (CFA modification indices)
RESI34	Overall, I am satisfied with myself.	Removed due to poor model fit (CFA modification indices)
RESI35	I believe I am valuable even if others disapprove.	Removed due to poor model fit (CFA modification indices)
RESI36	To change, I've distanced myself from people or situations that hurt me.	Excluded due to low factor loading (< 0.40) in EFA
RESI37	I believe distancing from some people can benefit me.	Excluded due to low factor loading (< 0.40) in EFA
RESI38	Recently, I've expressed helpful aspects of my personality.	Removed due to poor model fit (CFA modification indices)
RESI39	I recall and rely on my values to face challenges.	Retained in RED-5
RESI40	Positive traits like joy and sociability help me daily.	Removed due to poor model fit (CFA modification indices)
RESI41	I believe changing some eating habits could be good.	RED-11<br>F3 motivation to change Factor<br>Removed because it did not show satisfactory predictive validity
RESI42	Changing my eating habits scares me, but I still do it.	RED-11<br>F3 motivation to change Factor<br>Removed because it did not show satisfactory predictive validity
RESI43	I'm making some eating changes despite the anxiety.	RED-11<br>F3 motivation to change Factor<br>Removed because it did not show satisfactory predictive validity
RESI44	I'm motivated to change my eating despite difficulties.	Excluded due to low factor loading (< 0.40) in EFA
